# Acute Kidney Injury and Sepsis after Cardiac Surgery: The Roles of Tissue Inhibitor Metalloproteinase-2, Insulin-like Growth Factor Binding Protein-7, and Mid-Regional Pro-Adrenomedullin

**DOI:** 10.3390/jcm12165193

**Published:** 2023-08-09

**Authors:** Antonio Lacquaniti, Fabrizio Ceresa, Susanna Campo, Giovanna Barbera, Daniele Caruso, Elenia Palazzo, Francesco Patanè, Paolo Monardo

**Affiliations:** 1 Nephrology and Dialysis Unit, Papardo Hospital, 98158 Messina, Italy; 2 Cardiac Surgery Unit, Papardo Hospital, 98158 Messina, Italy; 3 Clinical Pathology Unit, Papardo Hospital, 98158 Messina, Italydanielecaruso@aopapardo.it (D.C.);

**Keywords:** acute kidney injury, TIMP2*IGBP7, mid-regional pro-adrenomedullin, sepsis

## Abstract

Background: Identifying a panel of markers detecting kidney injury before the glomerular filtration rate reduction is a challenge to improving the diagnosis and management of acute kidney injury (AKI) in septic patients. This study evaluated the roles of tissue inhibitor metal proteinase-2, insulin growth factor binding protein-7 (TIMP2*IGFBP7), and mid-regional pro-adrenomedullin (MR-proADM) in patients with AKI. Patients and Methods: This study was prospectively conducted in an intensive care unit (ICU) enrolling 230 patients who underwent cardiac surgery. Biomarkers were evaluated before and after 4 h of the cardiac surgery. Results: Whereas urine and creatinine alterations appeared at 23.2 (12.7–36.5) hours after cardiac surgery, urinary TIMP2*IGBP7 levels were higher at 4 h in AKI patients (1.1 ± 0.4 mg/L vs. 0.08 ± 0.02 mg/L; *p* < 0.001). Its concentration > 2 mg/L increases AKI risk within the following 24 h, clearly identifying the population at high risk of renal replacement therapy (RRT). In patients with sepsis, MR-proADM levels were 2.3 nmol/L (0.7–7.8 nmol/L), with the highest values observed in septic shock patients (5.6 nmol/L (3.2–18 nmol/L)) and a better diagnostic profile than procalcitonin and C-reactive protein to identify septic patients. MR-proADM values > 5.1 nmol/L and urine TIMP2*IGBP7 levels > 2 mg/L showed a significantly faster progression to RRT, with a mean follow-up time of 1.1 days. Conclusions: TIMP2*IGBP7 and MR-proADM precociously diagnose AKI in septic patients after cardiac surgery, giving prognostic information for RRT requirement.

## 1. Introduction

Acute kidney injury (AKI) is often related to sepsis in critically ill patients, secondary to the dysfunction of other organs or expression of the system-wide endothelial damage induced by hyper-inflammation and positive fluid balance [1].

Sepsis-induced AKI (sAKI) is a strong risk factor for mortality and adverse outcomes, and achieving a precocious diagnosis for suitable interventions is a major challenge in clinical practice to improve renal recovery and global patient outcomes.

A possible solution to this problem is to identify a marker or a panel of markers detecting kidney injury before the glomerular filtration rate (GFR) reduction, highlighting subclinical AKI compensated by the renal functional reserve that can be lost after a pathological noxa. 

The definition of AKI, according to KDIGO criteria, is based on serum creatinine and urinary output, neither sensitive nor specific, considering that many hours or days occur after the damage before their alterations and not evaluating potential variations depending on several patients’ characteristics. The consequent GFR reduction is not synchronous with the renal damage, and these two parameters vary with delay [2].

Numerous biomarkers have been investigated to predict AKI, such as neutrophil gelatinase-associated lipocalin (NGAL), tissue inhibitor metal proteinase (TIMP)-2, and insulin growth factor binding protein (IGFBP)-7, improving the diagnosis and giving prognostic information about the timing to start or stop a renal replacement therapy (RRT) [3,4,5]. In 2014, the American Food and Drug Administration and the European Medicines Agency approved the use of TIMP2*IGFBP7, urinary markers of cell cycle arrest reflecting cellular stress preceding tissue damage, to aid in the early prediction of AKI, with advantages in terms of diagnostic precocity and prognosis when compared to creatinine rise or urinary output reduction [6]. A diagnostic superiority in terms of sensitivity and specificity of this test was also revealed when compared to other renal biomarkers, such as urine or plasma NGAL, with a better prediction of moderate to severe AKI (KDIGO stage 2 to 3) in more than one thousand critically ill patients [7].

In the setting of sAKI, mid-regional pro-adrenomedullin (MR-proADM) is involved in capillary leakage, endothelial dysfunction, and multiple organ failure development [8]. MR-proADM is the precursor of the active form of adrenomedullin (ADM), a calcitonin peptide-like procalcitonin belonging to the calcitonin peptide family. MR-proADM is a stable fragment of 48 amino acids of the proADM molecule with a short half-life of 22 min, proportionally reflecting the ADM blood levels as a result of the split off from ADM in a 1:1 ratio [9]. ADM, produced predominantly by vascular endothelial cells, has a range of systemic biological actions including vasodilatation, increasing the cyclic adenosine monophosphate (cAMP) levels and the formation of nitric oxide, cell growth, regulation of hormone secretion, and antimicrobial effects [10]. 

At the renal level, its high expression in the glomeruli and tubules, assessed by immune-histochemical analyses, revealed diuretic and natriuretic actions through the tubules and vasodilatory actions, increasing GFR and the renal blood flow and dilating afferent and efferent arterioles [11]. Several studies demonstrated the relationship between MR-proADM levels, organ dysfunction, and endothelial damage severity [12,13]. High MR-proADM values characterized patients with sepsis, chronic kidney disease (CKD), or cardiac impairment due to myocardial infarction or chronic heart failure, with prognostic implications [11,14,15]. In CKD, ADM levels were markedly increased when compared with the normal controls and were related to the disease severity, irrespective of the basal renal disease [16]. Systemic inflammation and endothelial dysfunction characterized patients undergoing cardiac surgery. The hemodynamic instability, based on intravascular low volume, reduced cardiac preload, and anasarca state, promotes hypotension in a vicious circle and leads to shock and organ damage, such as AKI [17].

Starting from these assumptions, this study evaluated a panel of biomarkers including TIMP2*IGFBP7 and MR-proADM in patients with AKI that developed after cardiac surgery. Diagnostic properties were assessed as a precocious diagnosis of AKI if compared to creatinine and urine output. Prognostic information was also analyzed, evaluating the start of renal replacement therapy and the mortality rate in the intensive care unit.

## 2. Patients and Methods

### 2.1. Study Design

This observational prospective cohort single-center study was conducted in the intensive care unit (ICU) at Papardo Hospital, Messina, Italy, enrolling two hundred and thirty patients admitted to the ICU between January 2021 and December 2022 and undergoing coronary artery bypass graft surgery (CABG). Data were collected by the investigators and analyzed by external statisticians. The same experienced surgeons (F.P. and F.C.) have always performed surgery.

All patients underwent conventional open-heart surgery using aortic cross-clamping, intermittent hyperkalemic cardioplegic myocardial arrest, and cardiopulmonary bypass (CPB). In particular, a centrifugal pump (HL20 Heart-lung Machine, Maquet Getinge Group, Mississauga, ON, Canada) with a hollow-fiber membrane oxygenator (QUADROX i Adult, Maquet Getinge Group, Mississauga, ON, Canada) and a conventional air–oxygen blender were used in all the procedures. The venous reservoir was placed at a level of about 15 cm below the right atrium of the patient. Vacuum pressure was applied to the venous reservoir in order to achieve satisfactory venous drainage. The CPB target flow was 2.4 lt/min/m^2^ overflowing mainly in CKD patients and maintaining a mean arterial pressure of 85 mmHg in all patients. The CPB was mild hypothermic (32 °C) and the modification of blood gases was performed according to the pH stat model. 

After surgery, all patients were transferred to the cardiac surgery ICU. 

CRRT started when urine volume and/or serum creatinine values met the criteria for AKI diagnosis. Other indications were based on worsening of the patient’s condition associated with oliguria, severe hyperkalemia, and acidosis not responsive to intensive medical intervention, such as diuretics, volume expansion, and vasoactive agents. PrisMax^®^ (Baxter Healthcare Corporation, Chicago, IL, USA) and Multifiltrate Pro (Fresenius Medical Care, Bad Homburg, Germany) were the systems utilized, and the citrate represented the only method used for the regional anticoagulation.

The inclusion criteria were age ≥18 years and urinary catheter in place for at least 48 h, while exclusion criteria were age <18 years, patients with anuria or with diuresis less than 30 mL within 24 h of ICU admission, and a presumed life expectancy of fewer than 48 h after admission. Patients on hemodialysis or with a GFR < 15 mL/min were not enrolled. Moreover, patients were excluded if they underwent coronarography or radiologic procedures with iodinated contrast agents during the week before the surgery day. Patients who received extracorporeal membrane oxygenation (ECMO), percutaneous or surgical ventricular assist devices (VAD), or intra-aortic balloon pump (IABP) during or after the surgery were excluded from the study. 

### 2.2. Measurement of Biomarkers

Sera and urine samples for biomarker profiling evaluation were collected before the cardiac surgery and after 4 h from ICU admission.

All blood samples were stored at −80 °C, whereas urinary tests were immediately performed. All the biomarkers were measured at the Papardo Hospital laboratory by standard protocols in a technician-blinded manner. An additional blood sample was collected on the day of the diagnosis of sepsis and then stored at −80 °C to measure serum MR-proADM retrospectively.

Estimated GFR was calculated by the CKD Epidemiology Collaboration (CKD EPI) Equation [18]. 

### 2.3. Definitions

Clinical data recorded from the medical records included demographics, comorbidities, laboratories, and biomarker levels. 

CKD was classified according to the Kidney Disease: Improving Global Outcomes (KDIGO) 2012 Clinical Practice Guideline for the Evaluation and Management [19], whereas AKI was classified using the criteria in Kidney Disease: Improving Global Outcomes (KDIGO) [20]. 

Patients were followed up during the entire ICU stay and the Sequential Organ Failure Assessment (SOFA) score assessed the organ dysfunctions [21].

Sepsis or septic shock were defined according to the SEPSIS-3 consensus. In particular, sepsis was defined as life-threatening organ dysfunction caused by a dysregulated host response to infection. Organ dysfunction can be identified as an acute change in total SOFA score ≥ 2 points consequent to the infection. Patients with septic shock can be identified with a clinical construct of sepsis with persisting hypotension requiring vasopressors to maintain MAP ≥ 65 mm Hg and having a serum lactate level > 2 mmol/L (18 mg/dL) despite adequate volume resuscitation [22].

### 2.4. Outcomes

The primary endpoint was the diagnosis of AKI evaluating the TIMP2*IGBP7 levels and MR-proADM to identify septic patients. The secondary prognostic endpoint of MR-proADM was the start of renal replacement therapy and 30-day survival.

### 2.5. Statistical Analysis

Statistical analyses were performed with NCSS for Windows (version 4.0), the MedCalc (version 20.115; MedCalc Software Acacialaan, Ostend, Belgium) software, and the GraphPad Prism (version 9.4.1; GraphPad Software, Inc., San Diego, CA, USA) package. 

Differences between groups were established by unpaired *t*-test or by ANOVA followed by Bonferroni’s test for normally distributed values and by Kruskal–Wallis analysis followed by Dunn’s test for nonparametric values. Correlation coefficients were used as appropriate to test correlations between biomarkers and other variables. Before correlations were tested, all non-normally distributed values were log-transformed to better approximate normal distributions. 

A receiver operating characteristic (ROC) analysis was employed to calculate the area under the curve (AUC) for TIMP2*IGBP7 and MR-proADM to find the best cut-off values identifying AKI and septic status, respectively. Kaplan–Meier curves were generated to assess the progression to the endpoint, defined as the start of the renal replacement therapy (RRT) in subjects with serum MR-proADM and urinary TIMP2*IGBP7 values above and below the optimal ROC-derived cut-off levels. Differences were evaluated using the log-rank test.

The local Human Research Ethics Committee approved the study; the study complied with the Declaration of Helsinki.

## 3. Results

### 3.1. Baseline Characteristics of the Study Population

Table 1 summarizes the main baseline characteristics of the entire studied population. 

The mean age was 65.3 ± 7.9 years and 63% of patients were male; one hundred fifty-three patients (66%) had diabetes, whereas blood arterial hypertension was detected in one hundred sixty-six patients (72%). Eighty-nine (38%) subjects were active cigarette smokers, while chronic obstructive pulmonary disease (COPD) was revealed in ninety-four patients (41%). CKD was assessed in one hundred two subjects (44%) with a mean serum creatinine of 1.80 ± 0.9 mg/dL, determining a mean eGFR of 41.8 ± 18 mL/min (IQ range 20 to 55 mL/min). According to the baseline stages of GFR, 68 patients belonged to stage III, whereas 34 patients were included in stage IV. The mean number of grafts per patient was 2.4 ± 0.5. In the entire cohort, one hundred thirteen patients developed an AKI event during their stay in the ICU, including patients who had sepsis or septic shock. Among them, seventy-five patients were treated by RRT. 

### 3.2. TIMP2*IGBP7 and Diagnosis of AKI

Baseline TIMP2*IGBP7 levels were in the normal range in all enrolled patients at the pre-surgery evaluation (0.08 ± 0.02). These data were not influenced by pre-existing CKD, without differences assessed in patients with (0.10 ± 0.03 mg/L) or without renal disease (0.07 ± 0.02 mg/L), *p* > 0.05). While creatinine and urine output alterations did not occur until 23.2 (12.7–36.5) hours after cardiac surgery, urinary TIMP2*IGBP7 levels were higher at 4 h in patients who developed AKI when compared to baseline levels (1.1 ± 0.4 mg/L vs. 0.08 ± 0.02 mg/L; *p* < 0.001). Conversely, in patients without AKI, we did not reveal differences between the two measurements (0.12 ± 0.03 mg/L vs. 0.08 ± 0.02 mg/L; *p*: 0.12) (Table 2).

Furthermore, TIMP2*IGBP7 levels were associated with the severity of AKI, considering that a concentration > 2 mg/L assessed after 4 h from cardiac surgery increases the risk of KDIGO 3 AKI within the next 24 h and clearly identifying the population at high risk of AKI requiring renal replacement therapy (RRT). Conversely, values of this biomarker below 0.5 ruled out any need for renal replacement in the next 48 h of observation.

**Figure 1:**
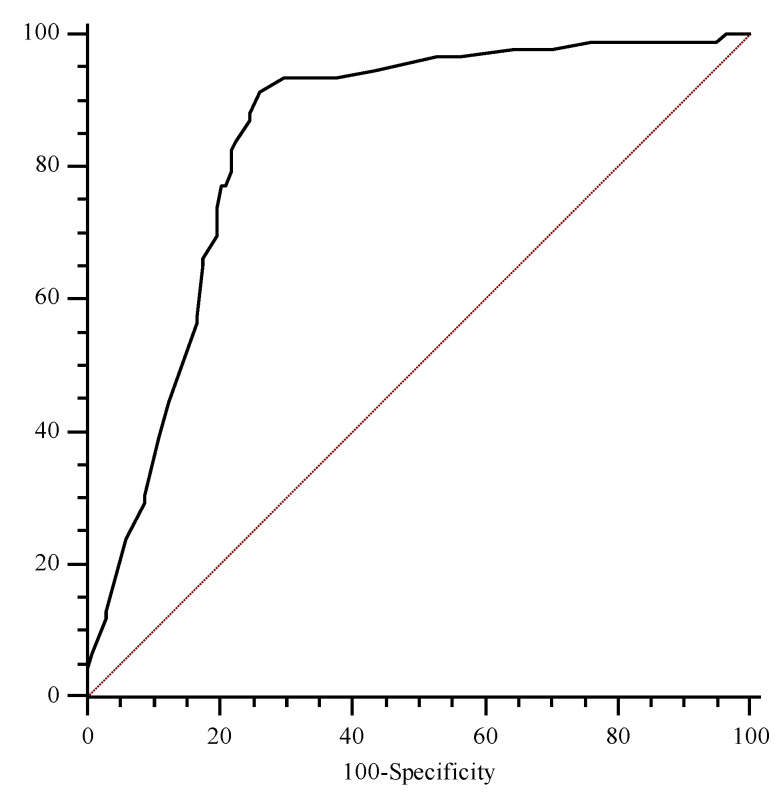
Receiver operating characteristic curves of tissue inhibitor metal proteinase-2 and insulin growth factor binding protein-7 (TIMP2*IGBP7) considering diagnosis of AKI as status variable.

The ROC analysis showed an AUC for this marker of 0.78 (95% CI, 0.68 to 0.85), with the best cut-off level found to be 2.0 mg/L (sensitivity 83.9%, specificity 73.8%) (Figure 1). 

### 3.3. Septic Patients

Sepsis was diagnosed in 83 patients (36%), including 21 subjects with septic shock. In particular, pneumonia represented the most common type of infection, involving 43 patients (52%) after cardiac surgery, detected by chest X-ray or computerized tomographic scanning and confirmed by microbiological assays. The microorganisms most commonly isolated were Enterobacteriaceae, Acinetobacter baumanii, and Pseudomonas aeruginosa. Surgical site infection of the sternal wound, and, with minor incidence, the limb site, involved eight patients (9%), with Staphylococci as the main bacteria assessed.

In six patients (7%), the catheters represented the suspected source of infection, assessed by microbiological tests. In the remaining 26 patients (32%) a nosocomial bloodstream infection represented the only datum related to the infection, without the identification of the source. Enterobacteriaceae and staphylococci represented the most common bacteria revealed. Moreover, combined sites of infection were observed in several septic patients. Patient characteristics with and without sepsis are shown in Table 3.

In septic patients, median MR-proADM levels were 2.3 nmol/L (0.7–7.8 nmol/L), whereas the highest values characterized patients with septic shock (5.6 nmol/L (3.2–18 nmol/L)). AKI was observed in 65% of septic patients, with the highest prevalence involving twenty-one patients with septic shock. In particular, 54 out of 83 patients (65%) were treated with RRT. In these patients, the highest mean values of TIMP2*IGBP7 were recorded after 4 h from cardiac surgery (3.2 ± 1.1 mg/L) and were associated with the highest values of MR-proADM. MR-proADM positively related with the SOFA score at ICU admission (r = 0.51; *p*: 0.001), procalcitonin (r = 0.63; *p* < 0.0001), C-reactive protein (r = 0.38; *p*: 0.01), and lactate (r = 0.49; *p*: 0.003), whereas this marker was inversely related to mean blood pressure (r = −0.28; *p*: 0.02) and eGFR (r = −0.41; *p*: 0.01). After the multivariate analysis, the correlation with the SOFA score (β: 0.31, *p*: 0.01), lactate (β: 0.401, *p*: 0.001), and mean blood pressure (β =−0.33; *p*: 0.01) remained significant. 

To define the optimal diagnostic cut-off for MR-proADM values to detect sepsis in our cohort, we performed an ROC analysis including only data from patients affected by sepsis or septic shock. The ROC analysis was performed with CRP and PCT. The AUC for MR-proADM on admission was 0.88 with the best cut-off at 5.1 nmol/L, determining a sensitivity and a specificity of 78.5% and 85%, respectively.

**Figure 2:**
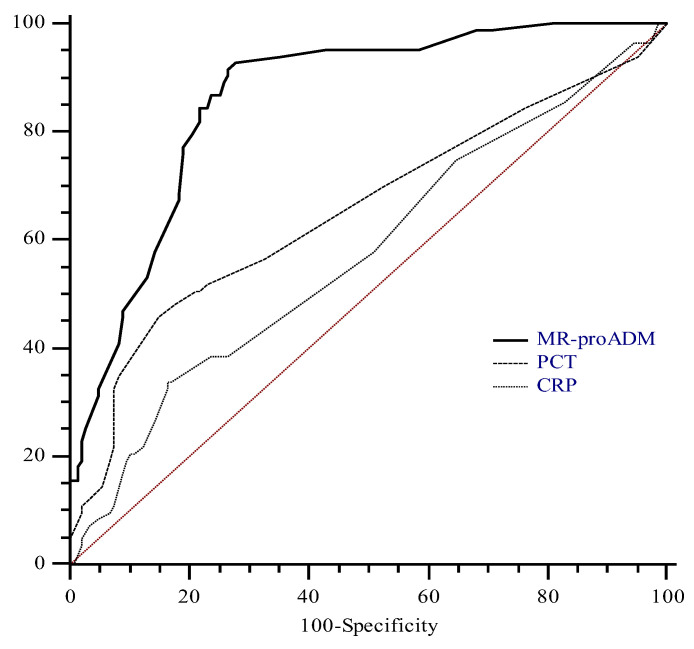
Receiver operating characteristics curves of mid-regional pro-adrenomedullin (MR-proADM), procalcitonin (PCT), and C-reactive protein (CRP) considering diagnosis of sepsis as status variable. MR-proADM area was significantly different from that of PCT (*p* < 0.0001) and CRP (*p* < 0.0001). On the contrary, the difference between PCT and CRP areas was not significant (*p*: 0.17).

The areas under the curve for PCT and CRP were 0.65 (95% CI, 0.59 to 0.71) and 0.57 (95% CI, 0.50 to 0.64), respectively. Both PCT and CRP areas were significantly different than that of MR-proADM (*p*: <0.001). On the contrary, the difference between the PCT and CRP areas was not significant (*p*: 0.17) (Figure 2).

### 3.4. Renal Replacement Therapy and AKI in Septic Patients

**Figure 3:**
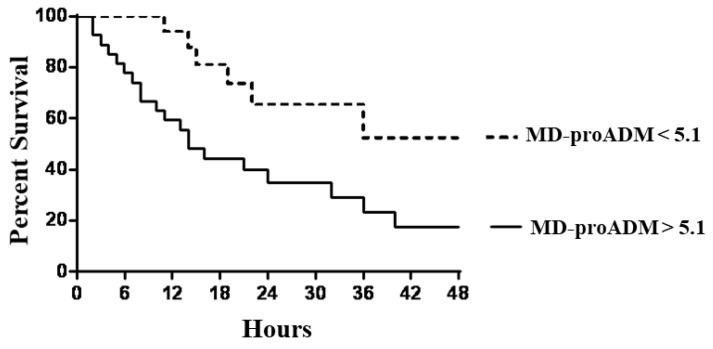
Kaplan–Meier survival curves of endpoints in patients with mid-regional pro-adrenomedullin (MR-proADM) levels above and below the optimal receiver operating characteristic cut-off level of 5.1 nmol/L.

We investigated the potential role of MR-proADM associated with TIMP2*IGBP7 to predict the necessity for RRT in septic patients. The ROC curve analysis revealed that MR-proADM at the cut-off > 5.1 nmol/L identified septic patients with a very good diagnostic profile. At the same time, AKI was well defined by a TIMP2*IGBP7 cut-off value > 2 mg/L. Subjects with MR-proADM values above 5.1 nmol/L experienced a significantly faster evolution to the endpoint, defined as the start of RRT (*p*: 0.002), with a mean follow-up time to progression of 2.5 days (95% CI, 1.1 to 4.6) compared with 9.2 days (95% CI, 6.3 to 12.1) for MR-proADM below the cut-off (Figure 3).

**Figure 4:**
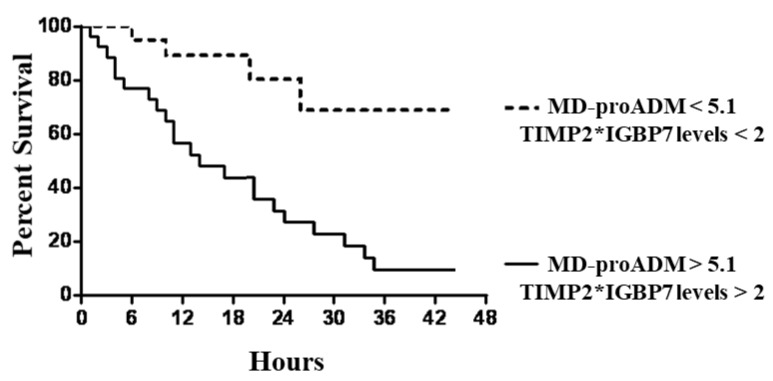
Kaplan–Meier survival curves of endpoint in patients with combined tissue inhibitor metal proteinase-2*insulin growth factor binding protein-7 (TIMP2*IGBP7) and mid-regional pro-adrenomedullin (MR-proADM) levels above and below the optimal receiver operating characteristic cut-off levels of 2 mg/L and 5.1 nmol/L.

Similar but stronger reports were evidenced if subjects were categorized according to MR-proADM values associated with TIMP2*IGBP7 levels. In particular, subjects with MR-proADM values above 5.1 nmol/L combined with urine TIMP2*IGBP7 levels > 2 mg/L showed significantly faster progression to the endpoint (*p* < 0.0001), with a mean follow-up time of 1.1 days (95% CI, 0.7 to 1.9) (Figure 4).

The 30-day all-cause mortality rate was very high in septic patients, involving 49 out of 83 subjects (59%). Sepsis-induced multiple organ failure (n = 20; 41%), septic shock (n = 18; 36%), and acute respiratory insufficiency (n = 11; 23%) represented the main causes of mortality. Higher concentrations of MR-proADM, PCT, and lactate as well as higher SOFA scores characterized non-surviving patients. This cohort experienced AKI events during the ICU stay, and all patients were treated by RRT. 

## 4. Discussion

This study demonstrated that urinary TIMP2*IGFBP7 represents a valid biomarker to highlight the high risk for AKI after cardiac surgery. Urinary values > 2.0 mg/L were strongly associated with AKI, with good sensitivity and specificity at 4 h after CABG predicting the requirement for RRT in critically septic patients and those with septic shock. These data strengthened the conclusions of a recent meta-analysis, showing a good diagnostic property of this biomarker characterized by an AUROC of 0.83 for the prediction of AKI within 24 h after cardiac surgery [23].

During the last two decades several biomarkers have been tested, but unfortunately, none of them are used today in clinical practice. This failure is related to their cost, availability, and issues related to low sensitivity or specificity. The complexity of the patients negatively influences the research in this field, which should be addressed through a combination of biomarkers. Moreover, different diagnostic criteria were used and too many methods were applied in various trials, reducing the possibility of obtaining uniform and valid conclusions, which could enhance the knowledge of physiopathology, incidence, outcomes, and risk factors for AKI. 

This study underlined that TIMP2*IGFBP7 was not influenced by a pre-existing CKD that often characterizes these patients, partially solving the issue observed for NGAL, the values of which were higher in urine or sera in CKD patients independently of AKI [24]. According to TIMP2*IGFBP7 levels, AKI was diagnosed 23 h before creatinine or urine output alterations. The precocity of the diagnosis is the key to nephrological management in the ICU, evaluating the patient at risk of renal damage from a multidisciplinary point of view, improving hemodynamic and fluid status to preserve an optimal kidney perfusion pressure, and avoiding nephrotoxic agents. The gold-standard definition of AKI, based on the KDIGO criteria, refers to kidney function, but not damage, with a consequence: the impossibility to detect structural, subclinical injury in functioning kidneys. A hybrid evaluation adding a panel of biomarkers could improve the diagnostic accuracy in these patients.

Moreover, a late AKI diagnosis could be the cause for the failure of several trials to reveal efficacy results after specific treatments, including drugs or RRT that failed to modify the disease course [25,26]. If the precocity of an AKI diagnosis improves the prognosis of postoperative cardiac surgery patients, this assumption is more suitable for sepsis. Early indicators of organ or immune dysfunctions are essential to start the most adequate therapies at the earliest opportunity [27,28,29].

The diagnosis of sepsis is based on two cornerstones: the SOFA score, highlighting the organ damages secondary to the septic process, and serum lactate, underlying tissue hypo-perfusion [22].

Our study assessed and confirmed that sepsis is a non-negligible clinical problem after cardiac surgery, with high prevalence and a negative impact on patient survival. We found a significant increase in MR-proADM in the plasma of septic patients compared with critical patients without infections, with an independent relationship with SOFA and lactate levels. Moreover, behind its diagnostic properties, its levels mirrored prognostic information relating to the severity of the septic status. The highest levels were recorded in patients with severe sepsis or septic shock requiring RRT, with better profiles than other classical biomarkers such as CRP or PCT. Furthermore, as a prognostic marker, MR-proADM levels were significantly higher in septic patients who did not survive than in survivors. Our data suggest the inclusion of MR-proADM evaluation into an early sepsis management protocol, permitting broader prognostic classification of septic patients, guiding early diagnostic interventions, and facilitating intensive treatment before any organ dysfunction.

Similar data were described by Elke, revealing that the initial use of MR-proADM within the first 24 h after sepsis diagnosis resulted in the strongest association with short-term, mid-term, and long-term mortality compared to all other biomarkers or clinical scores [8].

Our data confirmed the results obtained by the SISPCT trial that enrolled more than one thousand patients with severe sepsis and septic shock, demonstrating that MR-proADM more accurately predicted RRT requirement in the first week after ICU admission [30].

Recently, a meta-analysis revealed that MR-proADM was significantly and statistically higher among COVID-19 patients with a negative outcome, predisposing these subjects to an unfavorable prognosis [31].

The present study has some limitations that should be mentioned. First, it was a single-center study, and the cohort of patients was relatively small. Conversely, the single-center design guarantees the uniformity of the data collection and surgical technique. In the septic group, the generalizability of our findings is limited by the fact that only 83 patients were included. Confirmation in wider cohorts is indispensable to attribute general validity to our reports. Moreover, we did not evaluate MR-proADM as a precocious marker of treatment failure and did not evaluate its potential changes during ongoing antimicrobial therapy. Interventional studies to confirm these hypotheses are essential to identify non-responder patients who need alternative diagnostic and therapeutic interventions.

## 5. Conclusions

An associated evaluation of TIMP2*IGBP7 and MR-proADM precociously diagnoses AKI in septic patients after cardiac surgery, giving prognostic information for renal replacement therapy requirement and mortality risk.

## Figures and Tables

**Table 1:** Clinical and biochemical parameters of patients who underwent cardiac surgery at baseline.

	All Patients n: 230
Age, years	65.3 ± 7.9
Male n (%)	145 (63%)
BMI, kg/m^2^	31.5 ± 3.8
Hypertension, n (%)	166 (72)
Smoke, n (%)	89 (38)
Diabetes n (%)	153 (66)
COPD n (%)	94 (41)
CKD n (%)	102 (44)
LVEF > 50% n (%)	107 (46)
LVEF 30–50 n (%)	116 (50)
eGFR, mL/min	41.8 ± 18
sCreatinine, mg/dL	1.80 ± 0.9
TIMP2*IGFBP7, mg/L	0.08 ± 0.02

Abbreviations: BMI, body mass index; COPD, chronic obstructive pulmonary disease; CKD, chronic kidney disease; LVEF, left ventricular ejection fraction; CPB, cardiopulmonary bypass; eGFR, estimated glomerular filtration rate; sCreatinine, serum creatinine; TIMP2*IGFBP7, product of tissue inhibitor metalloproteinase-2 and insulin growth factor binding protein-7.

**Table 2:** Clinical, surgical, and biochemical parameters of patients after cardiac surgery, according to AKI.

	AKI Patients n: 113 (49%)	No AKI Patients n: 117 (51%)
Age, years	71.2 ± 4.6	67.8 ± 5.4
BMI, kg/m^2^	31.8 ± 2.1	30.4 ± 2.6
Hypertension, n (%)	79 (70)	97 (83)
Smoke, n (%)	39 (35)	50 (43)
Diabetes n (%)	77 (68)	76 (65)
COPD n (%)	53 (47)	41 (35)
CKD n (%)	71 (63)	31 (26)
LVEF >50% n (%)	44 (39)	63 (54)
CPB time, min	78.5 ± 21.2	69,2 ± 12.5
Aortic cross-clamping time, min	66.7 ± 23.4	56.7 ± 12.2
Operation time, min ± SD	179 ± 44	167 ± 54
TIMP2*IGFBP7, mg/L	1.1 ± 0.4	0.12 ± 0.03
RRT, n (%)	75 (66)	-
Sepsis, n (%)	54 (48)	29 (25)

Abbreviations: BMI, body mass index; COPD, chronic obstructive pulmonary disease; CKD, chronic kidney disease; LVEF, left ventricular ejection fraction; CPB, cardiopulmonary bypass; TIMP2*IGFBP7, product of tissue inhibitor metalloproteinase-2 and insulin growth factor binding protein-7; RRT, renal replacement therapy.

**Table 3:** Clinical and biochemical parameters of patients who developed AKI after cardiac surgery.

Parameter	Sepsis n: 83 (36%)	Non-Sepsis n: 147 (64%)
Age, years ± SD	68.9 ± 6.1	64.7 ± 5.2
Diabetes, n (%)	72 (87)	81 (55)
Hypertension, n (%)	79 (95)	87 (59)
CKD, n (%)	71 (85)	31 (21)
CPB time, min ± SD	85.3 ± 16.5	73.4 ± 15.2
Operation time, min ± SD	191.5 ± 32	167.2 ± 51
ICU LOS time, days, median (IQR)	19.7 (12.7–28.4)	7.4 (5.4–13.4)
ICU mortality, n (%)	49 (59)	13 (9)
AKI, n (%)	54 (65)	59 (40)
RRT, n (%)	54 (65)	21 (14)
MR-proADM, nmol/L, median (IQR)	2.3 (0.7–7.8)	-

Abbreviations: CKD, chronic kidney disease; CBP, cardiopulmonary bypass; LOS, length of stay; ICU, intensive care unit; AKI, acute kidney injury; CRRT, continuous renal replacement therapy; TIMP2*IGFBP7, product of tissue inhibitor metalloproteinase-2 and insulin growth factor binding protein-7; MR-proADM, mid-regional pro-adrenomedullin; SD, standard deviation; ICU mortality refers to 30 days of observation; min: minutes.

## Data Availability

The data associated with the paper are not publicly available but are available from the corresponding author on reasonable request.

## References

[1] Uchino S., Kellum J.A., Bellomo R., Doig G.S., Morimatsu H., Morgera S., Schetz M., Tan I., Bouman C., Macedo E. (2005). Acute renal failure in critically ill patients: A multinational, multicenter study. JAMA.

[2] Lacquaniti A., Caccamo C., Salis P., Chirico V., Buemi A., Cernaro V., Noto A., Pettinato G., Santoro D., Bertani T. (2016). Delayed graft function and chronic allograft nephropathy: Diagnostic and prognostic role of neutrophil gelatinase-associated lipocalin. Biomarkers.

[3] Bolignano D., Coppolino G., Romeo A., Lacquaniti A., Buemi M. (2010). Neutrophil gelatinase-associated lipocalin levels in chronic haemodialysis patients. Nephrology.

[4] Tao X., Chen C., Luo W., Zhou J., Tian J., Yang X., Hou F.F. (2021). Combining renal cell arrest and damage biomarkers to predict progressive AKI in patient with sepsis. BMC.

[5] Murray P.T., Mehta R.L., Shaw A., Ronco C., Endre Z., Kellum J.A., Chawla L.S., Cruz D., Ince C., Okusa M.D. (2020). ADQI 10 workgroup Potential use of biomarkers in acute kidney injury: Report and summary of recommendations from the 10th Acute Dialysis Quality Initiative consensus conference. Kidney Int..

[6] Godi I., Kashani K., Boteanu R., Martino F., Carta M., Giavarina D., Ronco C. (2021). Clinical adoption of Nephrocheck^®^ in the early detection of acute kidney injury. Ann. Clin. Biochem..

[7] Kashani K., Al-Khafaji A., Ardiles T., Artigas A., Bagshaw S.M., Bell M., Bihorac A., Birkhahn R., Cely C.M., Chawla L.S. (2013). Discovery and validation of cell cycle arrest biomarkers in human acute kidney injury. Crit. Care.

[8] Elke G., Bloos F., Wilson D.C., Brunkhorst F.M., Briegel J., Reinhart K., Loeffler M., Kluge S., Nierhaus A., Jaschinski U. (2018). The use of mid-regional proadrenomedullin to identify disease severity and treatment response to sepsis—A secondary analysis of a large randomised controlled trial. Crit. Care.

[9] Morgenthaler N.G., Struck J., Alonso C., Bergmann A. (2005). Measurement of midregional proadrenomedullin in plasma with an immunoluminometric assay. Clin. Chem..

[10] Hinson J.P., Kapas S., Smith D.M. (2000). Adrenomedullin, a multifunctional regulatory peptide. Endocr. Rev..

[11] Nishikimi T. (2007). Adrenomedullin in the kidney-renal physiological and pathophysiological roles. Curr. Med. Chem..

[12] Temmesfeld-Wollbrück B., Brell B., Dávid I., Dorenberg M., Adolphs J., Schmeck B., Suttorp N., Hippenstiel S. (2007). Adrenomedullin reduces vascular hyperpermeability and improves survival in rat septic shock. Intensive Care Med..

[13] Baldirà J., Ruiz-Rodríguez J.C., Wilson D.C., Ruiz-Sanmartin A., Cortes A., Chiscano L., Ferrer-Costa R., Comas I., Larrosa N., Fàbrega A. (2020). Biomarkers and clinical scores to aid the identification of disease severity and intensive care requirement following activation of an in-hospital sepsis code. Ann. Intensive Care.

[14] Christ-Crain M., Morgenthaler N.G., Struck J., Harbarth S., Bergmann A., Müller B. (2005). Mid-regional pro-adrenomedullin as a prognostic marker in sepsis: An observational study. Crit. Care.

[15] Maisel A., Mueller C., Nowak R., Peacock W.F., Landsberg J.W., Ponikowski P., Mockel M., Hogan C., Wu A.H., Richards M. (2010). Mid-region pro-hormone markers for diagnosis and prognosis in acute dyspnea: Results from the BACH (Biomarkers in Acute Heart Failure) trial. JACC.

[16] Nishikimi T., Horio T., Kohmoto Y., Yoshihara F., Nagaya N., Inenaga T., Saito M., Teranishi M., Nakamura M., Ohrui M. (2001). Molecular forms of plasma and urinary adrenomedullin in normal, essential hypertension and chronic renal failure. J. Hypertens..

[17] Hall R. (2013). Identification of inflammatory mediators and their modulation by strategies for the management of the systemic inflammatory response during cardiac surgery. J. Cardiothorac. Vasc. Anesth..

[18] Levey S.A., Stevens A.L., Schmid H.C., Zhang Y.P., Kusek J.W., Eggers P., Van Lente F., Greene T., Coresh J., Feldman, I.H. for the chronic kidney disease epidemiology collaboration (CKD-EPI) (2009). A new equation to estimate glomerular filtration rate. Ann. Intern. Med..

[19] Eknoyan G., Lameire N., Eckardt K., Kasiske B., Wheeler D., Levin A. (2013). KDIGO 2012 clinical practice guideline for the evaluation and management of chronic kidney disease. Kidney Int..

[20] Khwaja A. (2012). KDIGO Clinical Practice Guideline for Acute Kidney Injury. Kidney Int. Suppl..

[21] Vincent J.L., Moreno R., Takala J., Willatts S., De Mendonça A., Bruining H., Reinhart C.K., Suter P.M., Thijs L.G. (1996). The SOFA (sepsis-related organ failure assessment) score to describe organ dysfunction/failure. On behalf of the working group on sepsis-related problems of the european society of intensive care medicine. Intensive Care Med..

[22] Singer M., Deutschman C.S., Seymour C.W., Shankar-Hari M., Annane D., Bauer M., Bellomo R., Bernard G.R., Chiche J.D., Coopersmith C.M. (2016). The Third International Consensus Definitions for Sepsis and Septic Shock (Sepsis-3). JAMA.

[23] Su L.J., Li Y.M., Kellum J.A., Peng Z.Y. (2018). Predictive value of cell cycle arrest biomarkers for cardiac surgery-associated acute kidney injury: A meta-analysis. Br. J. Anaesth..

[24] Bourgonje A.R., Abdulle A.E., Bourgonje M.F., Kieneker L.M., la Bastide-van Gemert S., Gordijn S.J., Hidden C., Nilsen T., Gansevoort R.T., Mulder D.J. (2023). Plasma Neutrophil Gelatinase-Associated Lipocalin Associates with New-Onset Chronic Kidney Disease in the General Population. Biomolecules.

[25] Ruth A., Basu R.K., Gillespie S., Morgan C., Zaritsky J., Selewski D.T., Arikan A.A. (2021). Early and late acute kidney injury: Temporal profile in the critically ill pediatric patient. Clin. Kidney J..

[26] Zarbock A., Kellum J.A., Schmidt C., Van Aken H., Wempe C., Pavenstädt H., Boanta A., Gerß J., Meersch M. (2016). Effect of Early vs Delayed Initiation of Renal Replacement Therapy on Mortality in Critically Ill Patients with Acute Kidney Injury: The ELAIN Randomized Clinical Trial. JAMA.

[27] Bloos F., Rüddel H., Thomas-Rüddel D., Schwarzkopf D., Pausch C., Harbarth S., Schreiber T., Gründling M., Marshall J., Simon P. (2017). Effect of a multifaceted educational intervention for anti-infectious measures on sepsis mortality: A cluster randomized trial. Intensive Care Med..

[28] Andriolo B.N., Andriolo R.B., Salomao R., Atallah A.N. (2017). Effectiveness and safety of procalcitonin evaluation for reducing mortality in adults with sepsis, severe sepsis or septic shock. Cochrane Database Syst. Rev..

[29] Campo S., Lacquaniti A., Trombetta D., Smeriglio A., Monardo P. (2022). Immune System Dysfunction and Inflammation in Hemodialysis Patients: Two Sides of the Same Coin. J. Clin. Med..

[30] Bloos F., Trips E., Nierhaus A. (2016). Effect of sodium selenite administration and procalcitonin-guided therapy on mortality in patients with severe sepsis or septic shock: A randomized clinical trial. JAMA Intern. Med..

[31] Fialek B., De Roquetaillade C., Pruc M., Navolokina A., Chirico F., Ladny J.R., Peacock F.W., Szarpak L. (2023). Systematic review with meta-analysis of mid-regional pro-adrenomedullin (MR-proadm) as a prognostic marker in Covid-19-hospitalized patients. Ann. Med..

